# Virulence and Pathogenicity Properties of *Aggregatibacter actinomycetemcomitans*

**DOI:** 10.3390/pathogens8040222

**Published:** 2019-11-06

**Authors:** Georgios N. Belibasakis, Terhi Maula, Kai Bao, Mark Lindholm, Nagihan Bostanci, Jan Oscarsson, Riikka Ihalin, Anders Johansson

**Affiliations:** 1 Division of Oral Diseases, Department of Dental Medicine, Karolinska Institutet, S-141 04 Huddinge, Sweden; george.belibasakis@ki.se (G.N.B.); kai.bao@ki.se (K.B.); nagihan.bostanci@ki.se (N.B.); 2 Department of Biochemistry, University of Turku, FI-20014 Turku, Finland; terhi.maula@utu.fi (T.M.); riikka.ihalin@utu.fi (R.I.); 3 Department of Odontology, Umeå University, S-901 87 Umeå, Sweden; mark.lindholm@umu.se (M.L.); jan.oscarsson@umu.se (J.O.)

**Keywords:** *Aggregatibacter actinomycetemcomitans*, leukotoxin, cytolethal distending toxin, lipopolysaccharides, cytokine binding factors, horizontal gene transfer, outer membrane vesicles, biofilm, proteomic

## Abstract

*Aggregatibacter actinomycetemcomitans* is a periodontal pathogen colonizing the oral cavity of a large proportion of the human population. It is equipped with several potent virulence factors that can cause cell death and induce or evade inflammation. Because of the large genetic diversity within the species, both harmless and highly virulent genotypes of the bacterium have emerged. The oral condition and age, as well as the geographic origin of the individual, influence the risk to be colonized by a virulent genotype of the bacterium. In the present review, the virulence and pathogenicity properties of *A. actinomycetemcomitans* will be addressed.

## 1. Introduction

**Figure 1:**
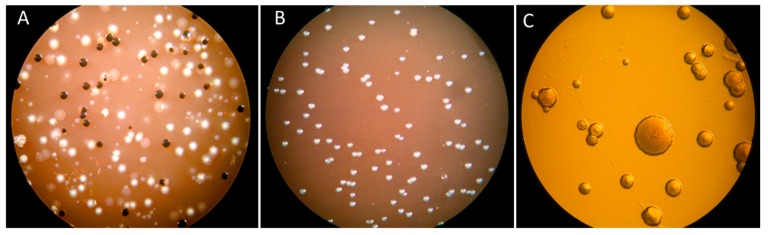
Subgingival biofilm samples from pathological periodontal pockets cultivated on blood agar plates for 48 h at 37 °C in an anaerobic atmosphere. (**A**) Sample obtained from a periodontal pocket of a middle-aged patient diagnosed with generalized chronic periodontitis. The macroscopical observation of the plated sample showed occurrence of a variety of colonies corresponding to different bacterial species. (**B**) Sample obtained from a periodontal pocket of a 25-year-old patient diagnosed with localized aggressive periodontitis. The macroscopical observation of the plated sample showed predominantly the occurrence a single colony type. (**C**) Microscopical (50× magnification) examination indicated that all colonies belonged to the *A. actinomycetemcomitans* species, which was confirmed in assays based on genetic characterization.

*Aggregatibacter actinomycetemcomitans* is a facultative anaerobic Gram-negative bacterium that expresses several virulence factors, which activates a host response that could be associated to the pathogenesis of periodontitis [1]. This review will elaborate in more detail the virulence properties of *A. actinomycetemcomitans* that contribute to the increased pathogenicity of this species, particularly with regard to early and rapidly progressive forms of periodontal disease [2,3], such as localized aggressive periodontitis, where it is frequently a predominant find (Figure 1). The “crown jewel” of the virulence factors of *A. actinomycetemcomitans* has long been its leukotoxin [4,5]. However, a cytolethal distending toxin (CDT) has also been identified, making this species the only member of the oral microbiome to produce these two, or any of the two, protein exotoxins [6]. Its lipopolysaccharide is quite special in that the immunological responses elicited by the host can be used in classifying (serotyping) the virulence identity of each one of its strains [7]. More recently identified cytokine-binding molecules add to its potential virulence factors, suggesting additional pathogenicity mechanisms by which it can manipulate the host [8]. *A. actinomycetemcomitans* is also equipped with a wealth of outer membrane vesicles, like all Gram-negative species, which might confer special virulence properties to this species [9]. There is a great genetic diversity within this species, with base composition biases in the genomic islands suggesting their acquisitions via horizontal gene transfer [10]. Recent advances in biofilm modeling and proteomic technologies have helped define the localization of *A. actinomycetemcomitans* within biofilms, characterize the full range of its protein components, and define how these are regulated by other species, and vice versa, when growing within complex polymicrobial communities [11]. Increased knowledge about bacterial virulence markers in periodontal disease may be important tools in future strategies for personalized dentistry [12].

## 2. Leukotoxin (LtxA)

**Figure 2:**
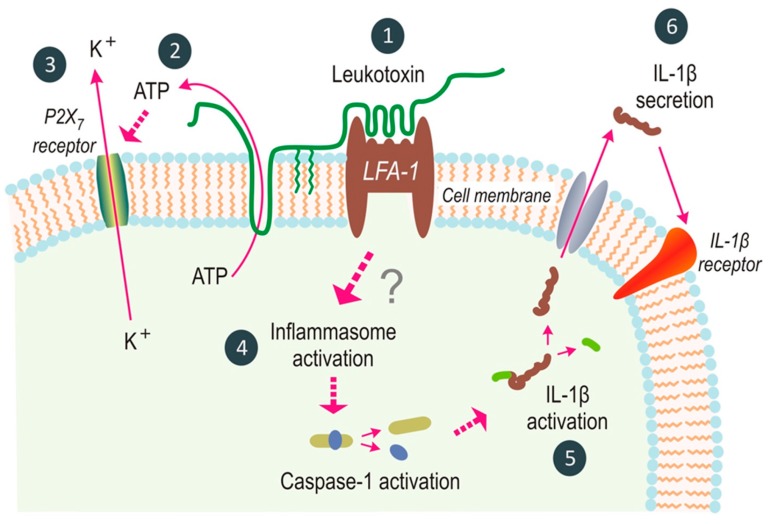
Leukotoxin (LtxA) induces a rapid inflammatory cell death in human macrophages. Briefly, LtxA binds to the LFA-1 receptor (1) and induces an extracellular release of ATP (2), which act as a ligand for the P2X7-receptor and result in an efflux of potassium (3). These processes promote the formation of an inflammasome multimer (4) that activates the cysteine proteinase caspase-1, resulting in a rapid activation (5) and secretion of IL-1β (6). Courtesy of Haubek and Johansson [20].

The leukotoxin (LtxA) of *A. actinomycetemcomitans* affects the different leukocyte populations with various death mechanisms [4]. It activates neutrophil degranulation causing a massive release of lysosomal enzymes, net-like structures, and matrix metallo proteinases (MMPs) and induces apoptosis in lymphocytes [13,14,15]. Interestingly, net-like structures can also be released from the LtxA-exposed neutrophils under anaerobic conditions and contain citrullinated proteins with sequence homology to proteins found in inflamed joints [16,17]. In the monocytes/macrophages, the toxin activates the inflammasome complex including the cysteine proteinase caspase-1, which induces an activation and secretion of the pro-inflammatory cytokines IL-1β and IL-18 [2,18,19]. These cellular and molecular mechanisms have been previously described in detail [20] (Figure 2). Taken together, several of the mechanisms by which LtxA affects leukocytes are also involved in the pathogenic mechanisms of many inflammatory disorders, such as periodontitis [21]. LtxA show a high target cell specificity expressed and affect only cells of hematopoetic origin from humans and some other primates [5]. This species-specificity of LtxA is reported to act through a unique target cell receptor and a specific region in the toxin that recognizes and interacts with this receptor [22,23]. A region of LtxA contains a series of 14 tandemly repeated amino acid sequences in the repeat region of the toxin and are shown to be responsible for the receptor binding to Lymphocyte function–associated antigen 1 (LFA-1) [4,23]. The LFA-1 molecule is a heterodimer consisting of the αL (CD11a) and β2 (CD18) subunits and is suggested to help the toxin to have a correct orientation on the target cell membrane [24,25,26,27].

### 2.1. LtxA Production

The LtxA operon belongs to the core genome of *A. actinomycetemcomitans* and is so far present in all examined strains [28]. The operon consists of four coding genes named *ltxC, ltxA, ltxB*, and *ltxD* and an upstream promoter region [29]. The gene *ltxA* encodes the LtxA protein, *ltxC* a protein required for the posttranslational acylation of LtxA, and *ltxB* and *ltxD* proteins needed for the transport of the LtxA to the bacterial outer membrane. For regulation of the LtxA expression, there is a promoter region located upstream of the *ltxC* gene, and genetic differences within this region result in different genotypes with various LtxA expression [10].

Zambon [30] reported that leukotoxicity of *A. actinomycetemcomitans* isolated from individuals with periodontitis was enhanced compared with isolates from individuals without periodontitis. Later, it was discovered that many of the isolates with enhanced leukotoxicity have been shown to have a different type of promoter in the leukotoxin operon [29]. A specific genotype (JP2) of *A. actinomycetemcomitans* with enhanced leukotoxicity has been shown to significantly correlate to disease onset in infected individuals [31,32]. The JP2 genotype was first identified by Brogan and co-workers [33] and was a serotype b isolate with a 530 base pair (bp) deletion in the *ltxCABD* promoter. Based on this finding, isolates with such a deletion in the promoter are named JP2 genotype, and those lacking this deletion are non-JP2 genotype [20,34]. The discovery of the JP2 genotype introduced a new terminology of high and low leukotoxic *A. actinomycetemcomitans* based only on the *ltx* promoter type [33,35]. The presence of the JP2 genotype is highly associated to adolescents of North and West African origin [31,36]. However, the JP2 genotype has recently been shown to also colonize individuals of other geographic origin, as confirmed by genotyping [37,38]. Alterations in the *ltx* promoter region are the most studied genotypes associated with enhanced LtxA expression. In addition to the JP2 genotype, an insertion of 886 bp, as well as a 640 bp deletion in the *ltx* promoter, has been discovered in *A. actinomycetemcomitans* isolates [39,40]. These three different genotypes are all associated with high virulence due to enhanced production of LtxA. Whether the deletions or insertions per se are causing the increased leukotoxin production is not entirely clear.

Enhanced leukotoxicity has been reported from serotype b isolates with an intact *ltx* promoter region, indicating a high production of the toxin [41]. These isolates represent a subgroup of serotype b with a high association with disease progression in the infected individual. Genetic characterization of this subgroup showed a genetic pattern with similarities to that of the JP2 genotype, and such strains are frequently carried by young individuals with periodontitis [2,38,42]. A specific genetic marker (*cagE*) correlates to *A. actinomycetemcomitans* with enhanced leukotoxicity, including the JP2 genotype and other virulent serotype b isolates [42,43]. Interestingly, the activity of LtxA has been reported to be involved in induction of systemic autoantibodies to citrulline, which are risk markers for rheumatoid arthritis [17,44]. In addition, LtxA has a crucial role in sepsis induced by bacteremia in an animal model [45].

### 2.2. Leukotoxin Secretion

The secretion of LtxA is mediated by a Type I secretion system in line with other proteins of the repeat in toxin (RTX family) expressed by several Gram-negative pathogens [46,47,48]. A unique property for LtxA among the RTX proteins is that the secreted toxin is reported to be associated to the bacterial outer membrane [47]. The export of the toxin to the bacterial outer membrane has been shown to require expression of three proteins—LtxB, LtxD, and TdeA—for export of the toxin to the bacterial outer membrane, and a fourth—LtxC—for the acylation [49]. In addition, the inner membrane protein MorC, which affects the outer membrane structure, has been reported to be necessary for efficient export of the toxin [50]. The localization of LtxA was found to be on the outside of the bacterial membrane and on vesicles associated to the outer membrane [51,52]. The responsible mechanisms for the association of LtxA to the membrane are still not fully clarified, and whether the secreted LtxA remains associated to the bacterial outer membrane or is released to the environment is a topic of controversy. A suggestion is that the hydrophobic domain of LtxA mediates the association to the bacterial membrane [27]. Ohta and co-workers [53,54] reported that LtxA could be released from the bacterial membrane by DNase or RNase treatment, which indicates involvement of electrostatic forces between the negatively charged nucleic acid and the positively charged LtxA. This phenomenon was later supported by the observation that LtxA on the bacterial membrane and on vesicles was released in hypertonic NaCl solutions [55]. LtxA was also released from the bacterial membrane in presence of serum proteins, which indicates involvement of competitive mechanisms [56]. Differences in the culture media have been shown to determine the distribution of the produced LtxA between the bacterial outer membrane and the culture supernatant [56,57,58]. Whether LtxA is associated with the bacterial membrane or released to the environment at the infected site in vivo is still not known. However, the serum mediated release of the LtxA [56], as well as the activation of a systemic immunogenic response [59,60], indicates a release of LtxA from bacteria growing in an in vivo oral biofilm.

The secreted LtxA has been shown to be efficiently inactivated by proteases and superoxide radicals [55,61,62]. In 1981, McArthur and co-workers reported that the activity of LtxA in interaction with polymorphonuclear leukocytes (PMNs) was enhanced in the presence of human serum [63]. This phenomenon could later be explained by the protective effect of the serum protease inhibitors on the proteolytic enzymes released from LtxA challenged PMNs that degrades the toxin [56,64].

### 2.3. Quantification of LtxA Production

The great genetic diversity of *A. actinomycetemcomitans* has resulted in various genotypes with substantially different virulence properties, i.e., LtxA production [10,28]. The expression of LtxA is also influenced by environmental factors, such as growth conditions and substrates [46]. Notably, an anaerobic culture condition enhances substantially the production of LtxA, which mirrors the condition in the periodontal pocket [65]. 

Due to the complex regulation of *ltxA* expression, the balance between membrane-associated and secreted toxin, as well as its sensitivity to proteolytic degradation, a gold standard for quantification of LtxA production, is still not available. The first attempt to quantify LtxA activity was to add bacteria to isolated leukocytes and determine cell death by trypan blue exclusion [66]. In a study by Zambon and co-workers [67], the leukocyte lysis method was used to discriminate between leukotoxic and non-leukotoxic strains. This study showed that the prevalence of more leukotoxic variants of *A. actinomycetemcomitans* was higher in young individuals with periodontitis than in older individuals with the disease or in periodontally healthy individuals.

Except for examining the leukotoxicity of the isolates, methods targeting gene expression or immunodetection have been developed [58,68]. These methods have been employed on a limited number of *A. actinomycetemcomitans* isolates and support findings from previous leukotoxicity determinations, with enhanced expression in the JP2 genotype [69,70]. To obtain a gold standard for quantification of leukotoxicity of isolated *A. actinomycetemcomitans* is one of several challenges for future research.

## 3. Cytolethal Distending Toxin (CDT)

The CDT family comprises a number of bacterial protein exotoxins that is expressed by several Gram-negative species. Due to its deleterious effects on the host, as revealed in various experimental models, CDTs are likely to be involved in the etiopathogenesis of the associated human infections. They can be described as genotoxins, as their main action is to elicit DNA damage upon the intoxicated host cells [71]. The CDT holotoxin consists of subunits CdtA, CdtB, and CdtC. While CdtA and CdtC subunits mediate the internalization of the CdtB into the cell, the latter is translocated to the nucleus, causing its deleterious effects on the host cells. This subunit is functionally homologous to deoxyribonuclease I, hence it can cause DNA damage. It is postulated that CdtB internalization occurs via a mechanism involving the recognition of cell membrane cholesterol by both CdtC and CdtB [72,73].

*A. actinomycetemcomitans* expresses a CDT and is the only known oral species with this property. An estimated 66% to 86% of its strains express a CDT, and its presence has been associated with the occurrence of periodontal disease [74]. It is very plausible that its pathogenic effects are related to its capacity to cause DNA damage, cell cycle arrest, and eventually apoptosis to the intoxicated cells. This has been shown in structural cells like gingival fibroblasts and periodontal ligament cells [75,76], gingival epithelial cells [77,78,79,80], or gingival tissue explants [79], denoting that it can compromise the structural integrity and homeostatic capacity of the tissues. The capacity of CDT to affect the gingival epithelium has also been shown in human gingival explants [81], as well as in vivo upon inoculation of the toxin on rat gingival tissue [82]. Cells of the immune system are also highly susceptible to the cell cycle–arresting and apoptotic action of CDT, as has been demonstrated in human T cell [83], B-cells [84], and mononuclear cells [85]. CDT may also subvert the phagocytic capacity of macrophages and subvert their cytokine producing capacity [85]. The deleterious effects of CDT on cells of the immune system denote an impairment of the local immunity, which may compromise the capacity of the periodontium to recognize and eliminate the bacterial challenge, be it *A. actinomycetemcomitans* or other microbial constituents of the biofilm community. 

Another potentially pathogenic mechanisms activated by CDT is the stimulation of pro-inflammatory and osteolytic cytokine production by the intoxicated host cells [86]. It has been shown that CDT can stimulate the production of pro-inflammatory cytokines by peripheral blood mononuclear cells, such as interferon (IFN)-γ, Interleukin (IL)-1β, IL-6, and IL-8, a virulence property potentially independent of the toxin’s deoxyribonuclease I activity [85]. However, *A. actinomycetemcomitans* can stimulate the production of several pro-inflammatory cytokines and regulate inflammasome expression irrespective of its CDT, as demonstrated by the use of the *CDT*-deletion strains [87,88]. An important virulence property of CDT is revealed by its capacity to induce the major osteolytic factor receptor activator of nuclear factor kappa-B ligand (RANKL). This is a crucial molecule that stimulates the differentiation of osteoclasts and, consequently, bone resorption in periodontitis [89]. It has been shown that CDT induces RANKL expression and production in periodontal connective tissue cells, such as gingival fibroblasts and periodontal ligament cells [90,91], as well as T-cells [92]. This implies that the CDT may increase the levels RANKL in the periodontal tissues and therefore potentiate bone destruction by this action. The induction of inflammatory and bone-destructive molecular cascades in the periodontium by CDT may well constitute an additional mechanism through which *A. actinomycetemcomitans* is involved in the etiopathogenesis of periodontitis. On the other side of the bone remodeling equilibrium, when CDT acts directly on pre-osteoclasts, it may also induce apoptosis and hinder their differentiation to osteoclastic cells, thereby contributing a dysbalanced bone remodeling equilibrium that leads to periodontal breakdown [93].

## 4. Lipopolysaccharide and Cytokine-Binding Factors

### 4.1. The Virulence Properties of A. actinomycetemcomitans Lipopolysaccharide

**Figure 3:**
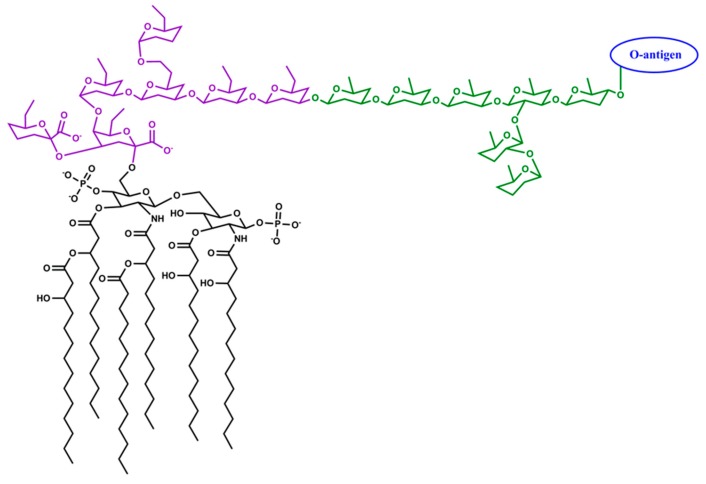
Schematic representation of a putative structure of the lipid A and the core oligosaccharides of *A. actinomycetemcomitans* lipopolysaccharide (LPS). The lipid A (black) of *A. actinomycetemcomitans* LPS is formed by four primary fatty acyl chains (myristic or 3-hydroxymyristic acid) linked by ester and amide linkages to a disaccharide of glucosamine. Two of the primary fatty acyl chains are esterified by secondary fatty acids. The acylation pattern of lipid A is asymmetric with four fatty acyl chains on the non-reducing glucosamine and two on the reducing glucosamine. The inner core (purple) is linked to lipid A by a ketosidic bond and is formed by 3-deoxy-D-*manno*-oct-2-ulosonic acid (Kdo) together with heptose residues such as *glycero*-*manno*-heptose. The outer core (green) usually consists of hexoses such as glucose and galactose. Functional groups such as hydroxyl and phosphate groups are common substituents in the lipid A and the core oligosaccharides. The O-specific polysaccharide chain (O-antigen) is the most variable portion in the LPS. The O-antigen consists of a large variety of sugar residues in many combinations and glycosidic linkages. For simplicity, substituents such as hydroxyl and phosphate groups (other than those in lipid A), conformational details of the monosaccharide residues, and the stereochemistry of the glycosidic bonds are not presented.

Like other Gram-negative species, *A. actinomycetemcomitans* surface is covered by lipopolysaccharide (LPS), a potent pro-inflammatory molecule. *A. actinomycetemcomitans* LPS comprises a group of structurally related molecules in which the O-specific polysaccharide chain (O-antigen), formed by oligosaccharide repeating units, is the most variable portion (Figure 3).

The serotyping of *A. actinomycetemcomitans* to seven different serotypes from a to f, as well as to non-serotypeable, is based on the structural differences in the O-antigen part of LPS. Commonly found monosaccharide residues in the *A. actinomycetemcomitans* O-antigen include the hexoses glucose, galactose, mannose, and talose; the hexosamines glucosamine and galactosamine; and the deoxyhexoses rhamnose and fucose. However, between different *A. actinomycetemcomitans* strains, there appears to be significant variation in the final architecture of the oligosaccharide repeating units, which may be either di-, tri-, or tetrasaccharide moieties [94,95,96,97]. Thus, variation in the gene clusters involved in the synthesis of the highly variable polysaccharide moieties serves as the basis for PCR serotyping of *A. actinomycetemcomitans* strains [94,98].

The structures of the core oligosaccharide and the lipid A in *A. actinomycetemcomitans* LPS have a lower degree of structural freedom than the O-antigenic polysaccharides. The core oligosaccharide consists of 3-deoxy-D-*manno*-oct-2-ulosonic acid (Kdo), *glycero*-*manno*-heptose, glucose, and galactose, and appears conserved among different serotypes [96,97]. The lipid A consists of two phosphorylated glucosamine residues [96,97] and primarily myristic and 3-hydroxymyristic acid as the fatty acyl chains [97,99,100,101,102]. Amino compounds such as ethanolamine and glycine are found associated with *A. actinomycetemcomitans* lipid A and core oligosaccharides but in fewer numbers than the commonly found hydroxyl and phosphate group substituents [97]. *A. actinomycetemcomitans* growth is favoured in slightly alkaline environments [103,104] in which the phosphate groups and the Kdo occur in deprotonated form. The phosphate groups and the Kdo interact with positively charged ions and participate in hydrogen bonding, and thus contribute to the stabilization of the bacterial outer membrane.

The fatty acyl composition of lipid A generally varies between different Gram-negative species. For example, in lipid A of *Escherichia coli* and *Salmonella typhimurium* LPS the relative amount of myristic acid is lower than in *A. actinomycetemcomitans* LPS, while lauric acid and palmitic acid are more frequently found [100]. The lipid A composition of closely related species may not always be specific enough to allow taxonomic differentiation, as demonstrated by the similar composition of *A. actinomycetemcomitans* and *Aggregatibacter aphrophilus* lipid A [100]. Closely related species may, however, be differentiated by the composition of their (core) oligosaccharides. A distinct feature of the oligosaccharides of *A. actinomycetemcomitans* is the presence of both D- and L-*glycero*-D-*manno*-heptose, whereas *A. aphrophilus* LPS only contains the L-enantiomer of this aldoheptose [105,106]. By contrast, galactose appeared twice as abundant in LPS from *A. aphrophilus* as in LPS from *A. actinomycetemcomitans* [99,105,106].

Although there are several studies that indicate a distinct effect of *A. actinomycetemcomitans* LPS on rodent cells [107,108,109,110,111], we will focus here on describing how *A. actinomycetemcomitans* LPS stimulates human cells. This outlining is done due to the known differences between the murine/rat and human immune systems [112]. The various virulence-related effects of *A. actinomycetemcomitans* LPS are summarized in Table 1. The first cells encountered by detached *A. actinomycetemcomitans* LPS in junctional epithelium (JE) of a tooth are the epithelial cells. These human epithelial cells have been shown to respond to *A. actinomycetemcomitans* LPS by expressing IL-15 which results in enhanced IFN-γ production and proliferation of human T cells [113]. Moreover, *A. actinomycetemcomitans* LPS causes widening of the intercellular spaces in primary tissue cultures mimicking the JE, a phenomenon not observed with *Porphyromonas gingivalis* LPS [113]. Various direct effects of *A. actinomycetemcomitans* LPS on the other main human gingival cell type, fibroblasts, have also been reported. Collagen is ingested and digested by gingival fibroblasts in balanced conditions of healthy gingiva. *A. actinomycetemcomitans* LPS is able to enhance the phagocytosis of collagen by fibroblasts which may result in imbalance in regeneration of the gingival tissue [114]. In addition to changes in cellular functions, *A. actinomycetemcomitans* LPS stimulate the production of IL-6 and IL-8 [114], tissue plasminogen activator (t-PA), and plasminogen activator inhibitor 2 (PAI-2) by human gingival fibroblasts [115]. The plasminogen/plasmin system is involved in the complex process of extracellular matrix degradation and renewal in the gingival tissue, a step that most likely precedes the collagen phagocytosis by the gingival fibroblasts.

Gingival tissue contains various resident immune cells, of which macrophages play a central role in periodontitis. *A. actinomycetemcomitans* LPS has shown to stimulate the expression of microRNA miR-146a [116], which downregulates the expression of TNF receptor–associated factor 6 and IL-1 receptor–associated kinase 1, which serves as a negative feedback loop in cytokine signaling [117]. On the contrary, high doses of *A. actinomycetemcomitans* LPS downregulated the expression of miR-32 and miR-29b, which target the pro-apoptotic factor Bim of acute myeloid leukemia cells [118] and IL-6 receptor α [117], respectively. Another human cell type that originates from mesenchymal stem cells is osteoblast which plays a significant role in bone formation. *A. actinomycetemcomitans* LPS has capacity to increase the inducible nitric oxide synthase (iNOS) activity and induce the nitric oxide (NO) production by human osteoblast-like cell line [119]. If osteoblasts produce NO rapidly, when responding to bacterial infection, it may lead to bone resorption [120].

Another type of resident immune cells in periodontium is dendritic cells (DCs), which are involved in antigen presenting to T cells. Conventional DCs originate from monocytes, like macrophages, and are called mDCs. Monocytes originating from localized aggressive periodontitis (LagP) patients, recently renamed molar-incisor pattern periodontitis with rapid progression [131], spontaneously give rise to mDCs [132]. *A. actinomycetemcomitans* LPS enhances the IL-12 production by mDCs leading to stimulation of IFN-γ expression of natural killer cells and undetectable levels of IL-4, which together may cause the polarization of naïve T cells toward the Th1 type response [123]. There are differences of DCs stimulation potential of *A. actinomycetemcomitans* LPS originating from different serotypes, serotype b LPS inducing the strongest production of IL-12, IFN-γ, TNF-α, IL-1β, IL-6, and IL-23 [124]. The differences in the response of DCs to serotype b *A. actinomycetemcomitans* LPS compared to the response to other serotype LPS most likely causes Th1/Th17 type of T cell response in serotype b related infection [133].

PMNs are innate immune cells which may play both defensive and destructive role in periodontitis [134]. Neutrophils produce reactive oxygen species (ROS) when responding to whole bacteria or their components. *A. actinomycetemcomitans* LPS has been shown to be more potent inducer of neutrophil ROS production than, for example, *P. gingivalis* or *Prevotella intermedia* LPS [125]. Moreover, *A. actinomycetemcomitans* LPS stimulate the production of inflammatory cytokines IL-1β and TNF-α by PMN more efficiently than *P. gingivalis* LPS [126], and in human whole blood, *A. actinomycetemcomitans* LPS causes higher production of the above mentioned cytokines as well as IL-6 than *E. coli* LPS [127]. *A. actinomycetemcomitans* LPS can shift the movement of monocytes and granulocytes from rolling to the passaging through the vascular wall by downregulating the surface expression of L-selectin and increasing the expression of β2 integrins, respectively [128].

Despite the vast literature concerning the inflammatory related functions of *A. actinomycetemcomitans* LPS, this outer membrane linked polysaccharide has also functions related to bacterial physiology. The serotype specific O-antigen part of *A. actinomycetemcomitans* LPS takes part in the secretion of leukotoxin, since a serotype b mutant with inactive *rmlC* and altered O-antigen sugar composition, contained more cytoplasmic and membrane-bound leukotoxin, and secreted less leukotoxin than the wild-type serotype b strain [135]. Moreover, the O-antigen part of *A. actinomycetemcomitans* LPS may mediate direct adhesion to abiotic surfaces [7]. Besides abiotic surfaces, *A. actinomycetemcomitans* LPS interacts with host molecules, such as human hemoglobin [129] and IL-8 [136], which may facilitate the iron acquisition and disturbance of host defense, respectively.

### 4.2. Sensing of Host Signal Molecules

*A. actinomycetemcomitans* is one of the human pathogens that is able to bind host cytokines, such as IL-1β, IL-8, and IL-6 [137,138], and internalize them [138,139], which leads to changes in the properties of biofilm, decreasing the metabolic activity [139], and changing the composition of the extracellular matrix [136,138]. Several potential bacterial proteins that may interact with human cytokines have been identified in *A. actinomyctemcomitans*, including intracellular ATP synthase subunit β [139], histone like DNA binding protein HU [139], and outer membrane proteins bacterial interleukin receptor I (BilRI) [138,140] and secretin channel HofQ [136]. The majority of these proteins have other functions in the bacterial cell related to metabolism, gene regulation, and uptake of nutrients and DNA for horizontal gene transfer, a character of so called “moonlighting” bacterial proteins [1]. Interactions with the above mentioned proteins may result in the observed uptake of cytokines, decreased metabolic activity and potentially, although not yet proved, also changes in the gene expression profile of *A. actinomycetemcomitans*. Both Gram-positive *Staphylococcus aureus* [141] and Gram-negatives *Pseudomonas aeruginosa* [142] and *Neisseria meningitidis* [143] are able to respond to cytokines, such as IL-1β, IFN-γ, IL-8, and TNF-α, by changing their virulence gene expression pattern.

Human cytokines are not the only host signalling molecules that *A. actinomyctemcomitans* is able to bind and sense. *A. actinomyctemcomitans* harbours the two-component system QseCB, which has originally been detected from enterohemorrhagic *Escherichia coli* EHEC [144]. In EHEC, QseCB senses either endogenous autoinducer-3 or host catecholamine hormones epinephrine/norepinephrine and induces the expression of Locus of Enterocyte Effacement (LEE) vital for the virulence of EHEC [145]. However, *A. actinomycetemcomitans* needs also iron, in addition to catecholamine hormone, to activate the QseC sensor kinase [146]. The QseCB signalling changes the gene expression pattern of *A. actinomycetemcomitans*, and especially the genes needed for anaerobic metabolisms are upregulated [146]. Moreover, QseC plays role in the biofilm formation in a flow cell in vitro model, since Δ*qseC* mutant strain forms significantly less biofilm in flow cells than the corresponding *A. actinomyctemcomitans* wild type and the complemented strains [147]. It is not known whether this impaired capacity to form biofilms also affects the virulence potential of *A. actinomyctemcomitans* in vivo. Yet, Δ*qseC* mutant strain causes less bone loss in murine model of periodontitis than the wild-type strain, suggesting a strong link between *A. actinomycetemcomitans* QseC and virulence [147].

## 5. Outer Membrane Vesicles

During the latest decades it has become apparent that membrane vesicles (MVs) are naturally shed during growth by many bacteria, archaea, and eukaryotes. Membrane vesicles, also known as “Type Zero secretion”, or referred to as outer membrane vesicles (OMVs) in Gram-negative organisms, serve as a general but relevant mechanism of antigen delivery, and are discharged by both commensal and pathogenic organisms in vivo and when infecting host cells in vitro [148,149]. Biologically active virulence factors such as CDT and OmpA can be transported into HeLa cells and human gingival fibroblasts via *A. actinomycetemcomitans* OMVs [150]. OMVs are also involved in the export of leukotoxin, peptidoglycan-associated lipoprotein (Pal), and the chaperonin GroEL to host cells [52,151,152,153]. Characterization of the OMV proteome of one *A. actinomycetemcomitans* clinical isolate using Matrix-Assisted Laser Desorption/Ionization Time of Flight-Mass Spectrometry (MALDI-TOF MS) revealed an array of additional tentative virulence-related proteins, including BilRI, Omp100, TdeA, and a ferritin-like protein [154,155]. This is in line with an OMV proteome exhibiting multiple offensive and defensive functions, such as drug targeting, iron acquisition, and immune evasion. A role of *A. actinomycetemcomitans* OMVs in serum resistance can be hypothesized based on observations that the vesicles could bind to the complement system regulator C4-binding protein in an OmpA-dependent manner [156]. Moreover, it has been shown that *A. actinomycetemcomitans* OMVs can transport small molecules such as those contributing to bone resorption, including LPS [157] and lipid A-associated proteins [158]. *A. actinomycetemcomitans* OMVs carry NOD1- and NOD2-active peptidoglycan, and upon vesicle internalization into non-phagocytic human cells such as gingival fibroblasts, the OMVs can act as an innate immunity trigger [9]. *A. actinomycetemcomitans* OMVs contain in addition nucleic acids [53], and recent evidence supports the concept that the OMVs can carry microRNA-sized small RNAs (msRNAs). These small RNAs might represent novel bacterial signaling molecules, which by means of OMVs can be transported into host cells to modulate the immune response [159]. *A. actinomycetemcomitans* OMVs appear to mainly internalize into host cells via clathrin-dependent endocytosis [9,160] but can also fuse with host cell membranes in a cholesterol-dependent manner [150]. Toxins associated with OMVs can function as adhesins in receptor-mediated endocytosis of vesicles [161], but such a role of CDT or leukotoxin appears less likely, as neither of the toxins are required for the uptake of OMVs into host cells [150,151]. Additionally, despite the evident localization of leukotoxin on the surface of *A. actinomycetemcomitans* OMVs, there is no requirement of the toxin receptor LFA1 for vesicle-mediated trafficking of LtxA into host cells [162].

## 6. Biofilm Interactions and Proteomic Regulations

### 6.1. Localization in Pocket and Tissue

As a microaerophic organism, *A. actinomycetemcomitans* is able to grow both supragingivally and subgingivally, corresponding to aerobic and anaerobic growth, respectively. As it can be detected at both locations, it has been postulated that the environment of supragingival plaque harbouring *A. actinomycetemcomitans* can act as a reservoir for the spread or reinfection of this bacterium of subgingivally [163]. When growing subgingivally, *A. actinomycetemcomitans* is reported to be detected in the loosely attached unattached plaque area in the middle pocket zone [164]. Earlier histopathological studies determined the prevalence and gingival localization of *A. actinomycetemcomitans* in periodontal lesions of juvenile periodontitis patients (earlier classification), using culture techniques on disrupted tissue or immunofluorescence microscopy on intact tissue. The former demonstrated the presence of *A. actinomycetemcomitans* in almost all diseased tissues examined, with evidence of microcolonies and single bacterial cells within the gingival connective tissue, as well as inside the phagocytic cells within the tissue [165]. The latter demonstrated an increase in *A. actinomycetemcomitans* colony-forming units, which correlated with its presence in the tissue and in the periodontal pocket [165]. In situ hybridization studies have detected *A. actinomycetemcomitans* in epithelial cells from the lining gingival crevice [166] or in close relationship with the polymorphonuclear infiltrate of the pocket [167]. By quantitative real-time PCR of gingival tissue lysates, it was shown that *A. actinomycetemcomitans* is present at a higher prevalence in tissues of younger patients with aggressive periodontitis as compared to chronic periodontitis or health [168].

### 6.2. Localization in Biofilms and Proteomic Interactions with Other Species

Studies on the localization of *A. actimomycetemcomitans* in biofilms largely comes from in vitro models. In supragingival and subgingival biofilms, *A. actinomycetemcomitans* does not appear to affect the number of the other species present and appears to form small, dense, and secluded cell clusters of its own species throughout the biofilm mass [169,170]. Yet, the lack of numeric changes on other species of the biofilm does exclude the possibility that *A. actinomycetemcomitans* exerts regulatory proteomic and metabolic changes in the biofilm, as discussed further.

The development of mass spectrometry (MS) technology made it possible to identify multiple proteins in a single run. Therefore, it is a useful tool to study how *A. actinomycetemcomitans* orchestrates proteomic changes in the context of biofilms. Using 2D gels, Llama-Palacios et al. discovered 87 protein spots differently expressed (1.5-fold, *p* < 0.05) between planktonic and mono-species biofilm cultures of *A. actinomycetemcomitans* [171]. Then, using MALDI-TOF MS, 13 upregulated proteins (from 24 proteins spots) and 37 downregulated proteins (from 50 spots) were identified. The upregulated proteins were mainly outer membrane proteins involved the immunologic process, whereas downregulated proteins were related to the metabolism, biosynthesis, and transport. This is consistent with the finding that mature biofilms display increased virulence [172] despite lower metabolic activity [173] as compared to planktonic culture.

Because oral biofilms occur as complex polymicrobial communities, the study of *A. actinomycetemcomitans* within multispecies biofilms is highly relevant. When integrated within a 10-species subgingival biofilm model, *A. actinomycetemcomitans* did not significantly impact the abundance of the other bacterial species, nor did it affect the biofilm structure, which is consistent with findings in a supragingival biofilm model. Using liquid chromatography–tandem mass spectrometry (LC-MS/MS), 3225 and 3352 microbial proteins were identified in multi-species biofilms in the absence or presence of *A. actinomycetemcomitans*, respectively [170]. Further investigations with label-free quantification (LFQ) method displayed 728 bacterial proteins and found 483 of them to be differentially regulated (2-fold, *p* < 0.05) among these two kinds of biofilms. Interestingly, the regulation trend for individual species was highly individual. For *Prevotella intermedia*, all quantified proteins were upregulated in the presence of *A. actinomycetemcomitans*, whereas the majority of the proteins were downregulated for *Campylobacter rectus*, *Streptococcus anginosus*, and *P. gingivalis*. These findings are well in line with the competing growth between *P. gingivalis* and *A. actinomycetemcomitans*, shown in a dual-species biofilm [174]. Furthermore, based on the GO analysis, *A. actinomycetemcomitans* appears to downregulate proteins with ferric iron binding functions and alter the metabolic rate for the overall biofilm.

To understand the effects of this *A. actinomycetemcomitans*-containing biofilms on host tissues, the biofilm model was introduced into a bioreactor-supervised 3D cell culture system, which consisted of epithelial and connective tissue structures to mimic the periodontium, as well as monocytes to stimulate the immune response [173]. As a result, *S. anginosus*, *A. oris*, *V. dispar*, *C. rectus*, and *P. gingivalis* were suppressed when present with the host tissue, while the tissue itself exhibited morphological, immunological, and proteomic changes. The numbers of *A. actinomycetemcomitans* in the biofilm were not reduced, but more of its proteins were expressed when co-cultured with the 3D (21 proteins) than the biofilm stand-alone (15 proteins) [173]. Yet, *A. actinomycetemcomitans* proteins only comprised a small fraction of all identified proteins in biofilm lysates (21 of 3363) and supernatants (one of 896).

Attachment to biotic surfaces or other biofilms enhances *A. actinomyctemcomitans* virulence properties. We have found that deletion of the gene *hns*, encoding a histone-like family of DNA-binding, nucleoid-structuring protein (H-NS), a global gene silencer [179], leads to a less piliated phenotype of *A. actinomycetemcomitans* and decreases its biofilm formation ability when cultured as mono-species biofilms [11]. LFQ showed that the majority (29) of the differentially expressed proteins (2-fold, *p* < 0.05) were upregulated in the *hns* mutant biofilm, supporting the role of H-NS as gene suppressor in *A. actinomyctemcomitans*. Notably, the affected proteins included virulence factors such as leukotoxin A and D (LtxA and LtxD). A similar repression activity of H-NS for virulence factors from other microbes was observed on hemolysin operons *hlyCABD* [180] and *ehxCABD* [181] in *E. coli* and *rtxACBD* in *Vibrio cholerae* [182]. Within multi-species biofilms, using the *hns* mutant there was a significant reduction of *A. actinomycetemcomitans* numbers, without affecting the number of other species. On the protein level, LFQ data suggested that many *Fusobacterium nucleatum* and *Streptococcus oralis* proteins were downregulated in biofilm harbouring the wild-type *A. actinomyctemcomitans* strain, as compared to its *hns* mutant counterpart, and these proteomic regulations may occur long before the corresponding bacterial growth is affected. Most of the regulated proteins were associated with peptide metabolic process and regulation of translation, supporting a protein-orchestrating role of H-NS in *A. actinomycetemcomitans*.

A literature summary of the proteomic findings on *A. actinomycetemcomitans* in single-species biofilms and multi-species biofilms is provided in Table 2 and Table 3, respectively.

## 7. Horizontal Gene Transfer

*A. actinomycetemcomitans* strains can be divided into competent and non-competent ones, which refer to their ability to acquire new genes from up-taken extracellular DNA (eDNA) using homologous recombination. Competent strains may have some advantage of being able to uptake eDNA, which could be related to additional means to repair DNA damage, obtain nucleotides and possibly also novel genes [184]. Approximately 30% of clinical *A. actinomycetemcomitans* strains are naturally competent [185], leading to greater genetic diversity, whereas noncompetent strains are genetically stable and need to use other mechanisms, such as conjugative plasmids, for horizontal gene transfer (HGT) [186]. It is thought that the ancestral Pasteurellacean was naturally competent and that noncompetent lineages lost their ability to take up DNA due to various mutations in the competence gene locus [187]. This may explain the findings that noncompetent strains are more common among some serotype groups, such as serotype b and c [185]. The competence gene locus consists of regulatory *sxy*, DNA uptake–related *comABCDE*, *pilABCD*, *comEAFE1*, *rec2*, and transformation-related *comM* and *urpA* [186,188,189]. Noncompetent strains may contain nonsense mutations, or insertions in one or several of these genes, which makes the strain either unable to uptake eDNA and/or to incorporate it into the genome [186]. In addition to the above described gene locus, the CRISPR-*cas* system is also closely involved in natural competence, since the loss of CRISPR-*cas* system is connected to the loss of competence [186]. Thus, non-competent *A. actinomycetemcomitans* strains are more prone to HGT caused by plasmids and phages. However, in the non-competent strains, the maintained CRISPRs contains few spacers that most likely are used for chromosomal gene regulation, since they possess specificity for endogenous genes [186].

The expression on genes of competence locus are regulated by *sxy* (*tfoX*), of which levels in the cell is affected by various environmental factors, such as extracellular calcium ions [190] and the biofilm mode of growth [190]. Moreover, in addition to competence locus and CRISPR-*cas* system, the development of competence and expression levels of *sxy* are stimulated by cyclic AMP [191] and affected by the *pga* gene cluster [190], respectively. The genes in *pga* cluster are needed in the production of most abundant extracellular polysaccharide, N-acetyl-D-glucosamine, in *A. actinomycetemcomitans* biofilms.

Naturally competent *A. actinomycetemcomitans* strains recognize related eDNA, which is suitable to be taken up, using uptake signal sequence (USS). The *Pasteurellaceae* family has two types of USS, *Hin* and *ApI*, which contain distinctive consensus sequences of nine base pairs. *A. actinomycetemcomitans* has *Hin* type USS, consisting of *A. actinomycetemcomitans* GTGCGGT consensus sequence followed by AT-rich repeats [187,191]. This sequence is most likely recognized by the outer membrane proteins involved in the eDNA uptake. However, the recognizing protein has not yet been identified.

## 8. Conclusions

*Aggregatibacter actinomycetemcomitans* is a facultative anaerobic Gram-negative bacterium with the capacity to employ many virulence mechanisms closely associated to the pathogenesis of periodontitis. The variety of virulence properties of this bacterium contribute to the pathogenicity of this species, particularly with regard to early and rapidly progressive forms of periodontal disease. *A. actinomycetemcomitans* can be found in a large proportion of the human population, and due to the large genetic diversity of this species, several different genotypes or phenotypes with various virulence properties have emerged. Without doubt, individuals carrying *A. actinomycetemcomitans* genotypes with a proven enhanced leukotoxin production have a significantly increased risk to develop disease. More recent studies have identified suitable genes of this species, which can potentially be traced as markers for epidemiological population monitoring and utilization in individual risk assessment programs. Such bacterial virulence markers in periodontal disease may prove to be important tools in future strategies for personalized dentistry.

## Figures and Tables

**Table 1:** Virulence-related properties of *A. actinomycetemcomitans* LPS.

Target	Effect	Reference
Epithelial cells	IL-15 expression, widening of the intercellular spaces	[113,121]
Gingival fibroblasts	Enhancement of collagen phagocytosis, increase the production of IL-8, IL-6, t-PA, PAI-2	[114,115,116,122]
Macrophages	Upregulation of miR-146a and downregulation of miR-32 and miR-29b microRNAs	
Osteoblasts	Increases iNOS activity and induce NO production	[119,123,124]
Dendritic cells	Production of IL-12, IFN-γ, TNF-α, IL-1β, IL-6, and IL-23	
PMN	Induce ROS production, stimulate IL-1β, TNF-α, and IL-6 production, downregulate surface expression of L-selectin, upregulate the expression of β2-integrins	[125,126,127,128]
Human hemoglobin	Binding	[129]
Human IL-8	Binding	[130]

**Table 2:** Proteomic studies on single species *A. actinomycetemcomitans.*

Author	Year (Ref)	Brief Description	Identified Proteins *	Proteomic Application, Label Free Quantification	Cutoff	PMID
Llama-Palacios et al.	2017 [171]	*A.a* planktonic and mono-species biofilm cultures	50	2DE, MALDI-TOF MS	N/A	28707473
Kieselbach T et al.	2016 [155]	*A.a* outer membrane vesicles dataset	501	In solution digestion, LC-MS/MS	protein FDR < 1%	28050585
Kieselbach T et al.	2015 [154]	*A.a* outer membrane vesicles	151	In solution digestion, LC-MS/MS	protein FDR < 1%	26381655
Smith KP et al.	2015 [175]	*A.a* membrane proteins related to morphogenesis protein C	613	Stable-isotope dimethyl labeling, nanoscale LC-MS	FP < 1%	25684173
Smith KP et al.	2015 [176]	*A.a* membrane proteins	648	Stable-isotope dimethyl labeling, nanoscale LC-MS	FP < 1%	25055881
Zijnge V et al.	2012 [177]	*A.a* secreted proteins from mono-species biofilm	179	2DE, HCT-Ultra ETD II IT-MS	Peptide ion score > 30	22848560
Rylev M et al.	2011 [178]	*A.a* JP2 strain HK1651	114	2DE, MALDI-TOF MS	N/A	21867783

Ion trap: IT, False positive: FP, False-discovery rate: FDR, Matrix assisted laser desorption ionization: MALDI, Time of flight mass spectrometry: TOF MS, Mass spectrometer: MS. * Maximum identified/quantified proteins were report base on the following rules: (1) Only maximum identified protein number was reported if the experiment was done under different conditions. (2) Total protein numbers were reported if the experiment has replicates. (3) The number of identified and quantified proteins were reported if only regulated protein were reported.

**Table 3:** Proteomic studies on multi-species biofilms including *A. actinomycetemcomitans.*

Author	Year (Ref)	Brief Description	Identified Proteins *	Proteomic Application	Peptide Cutoff	PMID
Bao et al.	2018 [169]	*A. a hns* + 10 species biofilms **	3352	Orbitrap Fusion, LFQ	2≥	25483866
Bao et al.	2015 [183]	10-species biofilm model * Vs 3D culture **	3363	Q-Exactive MS, LFQ	2≥	26525412
Bao et al.	2015 [170]	*A. a* + 10 species biofilms **	3352	Q-Exactive MS, LFQ	2≥	25756960

*A.a*: *A. actinomycetemcomitans*, Label free quantification: LFQ, Matrix-assisted laser desorption ionization- time of flight mass spectrometry: MALDI-TOF MS, Two-Dimensional Differential Gel Electrophoresis: 2DE, * Maxium identified/quantified proteins were report base on the following rules: (1) Only maximum identified protein number was reported if the experiment was done under different conditions. (2) Total protein numbers were reported if the experiment has replicates. (3) The number of identified and quantified proteins were reported if only regulated protein were reported. ** 10-species biofilm model (consisting of *Campylobacter rectus*, *Fusobacterium nucleatum*, *Porphyromonas gingivalis*, *Prevotella intermedia*, *Tannerella forsythia*, *Treponema denticola*, *Veillonella dispar*, *Actinomyces oris*, *Streptococcus anginosous*, *Streptococcus oralis*).
